# Perspectives on enhancing peer support services for mothers living with HIV in Kenya: A qualitative assessment of Mentor Mothers and the mothers they serve

**DOI:** 10.1371/journal.pgph.0007277

**Published:** 2026-09-18

**Authors:** James G. Carlucci, Rosa Chemwey Ndiema, Violet Naanyu, Marylydia Atieno Kiano, John M. Humphrey, Bridget Hawryluk, Edwin Were, Alan McGuire, Kara Wools-Kaloustian

**Affiliations:** 1 Department of Pediatrics, Indiana University School of Medicine, Indianapolis, Indiana, United States of America; 2 Department of Reproductive Health, Kenyatta National Hospital, Nairobi, Kenya; 3 Department of Sociology, Psychology and Anthropology, Moi University School of Arts and Social Sciences, Eldoret, Kenya; 4 Academic Model Providing Access to Healthcare (AMPATH), Eldoret, Kenya; 5 Department of Medicine, Indiana University School of Medicine, Indianapolis, Indiana, United States of America; 6 Research Jam, Indiana Clinical and Translational Sciences Institute, Indianapolis, Indiana, United States of America; 7 Department of Reproductive Health, Moi University College of Health Sciences, Eldoret, Kenya; 8 Department of Psychology, Indiana University Indianapolis, Indianapolis, Indiana, United States of America; 9 Health Services Research and Development, Richard L. Roudebush Veterans' Administration Medical Center, Indianapolis, Indiana, United States of America; Washington University in St. Louis, UNITED STATES OF AMERICA

## Abstract

Mentor Mother (MM) services are an evidenced-based peer support strategy embedded within prevention of mother-to-child transmission of HIV (PMTCT) programs. The current MM strategy in Kenya provides monthly, facility-based, one-size-fits-all antiretroviral therapy adherence counselling and health education to women living with HIV (WLHIV) who are navigating PMTCT services. MMs receive a small monthly stipend as compensation for providing these services. We aimed to gather formative information from MMs and their client WLHIV, with the goal of developing an enhanced MM strategy that accounts for the unique needs of each WLHIV. From April-July 2023, we conducted qualitative interviews with MMs (n = 16) and WLHIV (n = 36) from three public health facilities supported by Academic Model Providing Access to Healthcare (AMPATH) in western Kenya. Interviews and analysis were guided by the Practical, Robust Implementation and Sustainability Model (PRISM). Key findings included that both MMs and client WLHIV had favorable opinions about the possibility of developing and implementing a risk-stratified MM strategy wherein client WLHIV with identifiable risk factors receive intensified, problem-focused services while low-risk WLHIV are eligible for less intensive services. MMs and client WLHIV also agreed that empathetic MM-client relationships that leverage shared lived experiences to build trust and address stigma should remain central to the MM program. However, MMs were clear that a better-defined scope of work and capacity building through training and skills development would be necessary for successful implementation of an enhanced MM strategy. Additionally, motivating MMs with fair compensation and recognition for their work, as well as providing more resources for client WLHIV with economic barriers to care, could promote adoption and sustainability of an enhanced MM strategy. Additional participatory research, as well as input from system-level stakeholders, is needed to ensure feasible enhancements to MM services are contextualized within the real-world constraints of PMTCT programs.

## Introduction

There are more than 1 million pregnancies among women living with HIV (WLHIV) every year, and in many regions of Africa vertical transmission rates still exceed 5% [1]. In 2023, Kenya had an HIV prevalence of 3% among adults, but HIV prevalence was approximately twofold higher in women compared to men, and the vertical transmission rate was 7% [1]. Prevention of mother-to-child transmission of HIV (PMTCT) services, which start in antenatal care and continue through cessation of breastfeeding and definitive infant HIV testing, are the cornerstone to achieving optimal outcomes for WLHIV and their infants.

Mentor Mothers (MMs) are WLHIV who have previously navigated the PMTCT cascade then are recruited by the health system to serve as peer supporters for WHLIV currently enrolled in PMTCT services. MMs can provide support to WLHIV across a range of behavioral, social, and structural domains [2]. However, the extent to which MMs impact key clinical outcomes such as retention in care, viral suppression, infant uptake of diagnostic testing, and vertical transmission has varied across diverse contexts, between which MM strategies vary considerably [3–9]. For example, there are both facility-based [4,6,7] and community-based MM models [3,6,8,9], and there has been some prior research into utilizing MMs to provide structured peer support [4,7,8] and individualized counselling for client WLHIV [7]; however, there is a paucity of data about MM workload constraints [5], and research related to MM roles in risk-stratified differentiated service delivery models for PMTCT services are lacking.

In Kenya, MMs are typically health facility-based, and they encounter all WLHIV engaged in PMTCT services at each of their monthly antenatal and postnatal clinic visits, standardized in accordance with Ministry of Health (MoH) guidelines [10]. At each monthly encounter, MMs provide antiretroviral therapy (ART) adherence counselling and health education corresponding to PMTCT milestones (e.g., maternal viral suppression, facility-based delivery, exclusive breastfeeding, infant HIV testing, etc.). MMs also work with the clinic outreach team to make phone calls and home visits if WLHIV miss scheduled visits or are lost to follow-up, but MMs do not otherwise provide community-based services in this setting. MMs receive a small monthly stipend as compensation for providing these services. Current MM services do not include any formal risk stratification or differentiated service delivery, but there is some evidence that pregnant and postpartum Kenyan WLHIV prefer and could benefit from a more individualized and patient-centered approach than the current “one-size-fits-all” model [11–13]. Individualized care refers to tailoring interventions to a patient’s specific characteristics and needs, whereas patient-centered care more broadly emphasizes care that is responsive to patients’ values, preferences, and life circumstances.

The objective of this study was to gather formative information from MMs and their WLHIV clients, with the goal of developing an enhanced MM strategy that accounts for both the unique needs of each WLHIV and the real-world fiscal and human resource constraints of PMTCT programs. Specifically, we conducted qualitative interviews with MMs and their clients to assess perceptions of existing MM services, opportunities for feasible enhancements, barriers to optimizing the MM strategy, and approaches to overcoming these barriers.

## Methods

### Ethics statement

The study was approved by the Moi University/Moi Teaching and Referral Hospital Institutional Research and Ethics Committee in Kenya (IREC 332/2022) and the Indiana University Institutional Review Board in the United States (IRB 15648). A research permit was also granted by the National Commission for Science, Technology, and Innovation in Kenya (NACOSTI/P/23/22821).

### Setting

Data were collected from 04 April through 17 July 2023 at three Academic Model Supporting Access to Healthcare (AMPATH) supported public health facilities in western Kenya. AMPATH is a consortium with sentinel partnerships in Kenya, Ghana, Mexico, and Nepal [14]. AMPATH-Kenya is based in Eldoret, Uasin Gishu County and serves the surrounding region of western Kenya, including provision of HIV care and treatment for over 150,000 clients across the network of more than 300 care sites supported by the Kenyan MoH [15]. The three sites included in this study were hospital-based health facilities, each with inpatient services and adjacent outpatient clinics providing integrated HIV and maternal-child health services supported by facility-based MMs. Sites were purposively selected to capture variation in facility level and geographic setting, with additional consideration of feasibility, including existing collaborations with PMTCT teams and available resources. These sites include one level 6 national referral hospital, one level 5 county hospital, and one level 4 sub-county health facility, serving urban, peri-urban, and rural populations. The number of MMs at each facility is proportionate to the volume of WLHIV receiving care at each facility, and the structure and content of MM services is consistent across sites in accordance with MoH guidelines [10].

### Participants

All MMs from the three clinics (N = 16; Facility A [n = 7], Facility B [n = 6], and Facility C [n = 3]) were educated about the study then invited to participate. Postpartum WLHIV who were recipients of MM services at each of the three facilities, at least 15 years of age, and able to understand and provide informed consent in English or Kiswahili were eligible for inclusion. A purposive criterion-based sampling approach was used to obtain diverse perspectives from 36 WLHIV. Sampling of WLHIV was stratified by the following criteria: (i) clinic (Facility A vs. Facility B vs. Facility C); (ii) recency of HIV diagnosis (“new positives” vs. “known positives”); and (iii) PMTCT outcomes (favorable vs. unfavorable) (Table 1). We aimed for equal representation across strata to ensure adequate representation of each subgroup. New positives were defined as WLHIV diagnosed with HIV during their most recent pregnancy, while known positives were diagnosed with HIV prior to their most recent pregnancy. WLHIV were classified as having favorable outcomes if they had sustained viral suppression (i.e., consistently undetectable HIV viral loads throughout their most recent pregnancy and postpartum while breastfeeding), retention in care (i.e., no issues with missing appointments and not classified as lost to follow-up during their most recent pregnancy and postpartum while breastfeeding), and their infant received HIV testing on schedule and all infant HIV tests were negative. WLHIV were classified as having unfavorable outcomes if they had viremia (i.e., any detectable HIV viral load during their most recent pregnancy or postpartum while breastfeeding), lapses in care (i.e., issues with missing appointments or classification as being lost to follow-up during their most recent pregnancy or postpartum while breastfeeding), or their infant had tested positive for HIV or their HIV status was unknown due to not being tested on schedule. Only postpartum WLHIV were recruited to ensure that participants could speak to both antenatal and postpartum MM services and to support stratification by favorable and unfavorable outcomes, some of which could only be determined in the postpartum period (i.e., uptake of infant HIV testing and vertical transmission).

**Table 1:** Sampling strategy.

Study Site	Mentor Mothers(all Mentor Mothers from each facility)	Women living with HIV			
		New positives ^a^		Known positives ^b^	
		Favorable outcomes ^c^	Unfavorable outcomes ^d^	Favorable outcomes ^c^	Unfavorable outcomes ^d^
Facility A	7	3	3	3	3
Facility B	6	3	3	3	3
Facility C	3	3	3	3	3
**Total**	**16**	**36**			

^a^New positives = Newly diagnosed with HIV during their most recent pregnancy.

^b^Known positives = Known to have HIV prior to their most recent pregnancy.

^c^Favorable outcomes = Sustained viral suppression (consistently undetectable HIV viral loads throughout their most recent pregnancy and postpartum while breastfeeding), retention in care (no issues with missing appointments and not classified as lost to follow-up during their most recent pregnancy and postpartum while breastfeeding), *and* infant has been tested for HIV on schedule and all infant HIV tests have been negative.

^d^Unfavorable outcomes = Viremia (any detectable HIV viral load during their most recent pregnancy or postpartum while breastfeeding), lapse in care (issues with missing appointments or classification as being lost to follow-up during their most recent pregnancy or postpartum while breastfeeding), *or* infant has tested positive for HIV or their HIV status is unknown due to not being tested on schedule.

Clinical staff at the respective sites reviewed medical records to identify potentially eligible WLHIV, then a research assistant consecutively recruited eligible WLHIV at the conclusion of routinely scheduled clinic visits. WLHIV identified as having unfavorable outcomes due to being lost to follow-up, and who therefore could not be recruited directly from the clinic, were contacted by phone in partnership with the clinic’s outreach team and in accordance with AMPATH’s well established patient tracing protocols. Those who were contacted were invited back to the clinic or a mutually agreeable venue where recruitment could be completed.

### Data collection

A qualitative interview guide (S1 Text) was developed to facilitate in-depth assessments of MMs’ and client WLHIV’s perceptions of current MM services, desires for enhancements to the current MM strategy, and barriers and facilitators to optimizing the MM strategy. Development of the guide was informed by prior research about MMs and factors influencing PMTCT outcomes [2–9,11,16–20], discussions with PMTCT clinicians within the AMPATH program, and the Practical, Robust Implementation and Sustainability Model (PRISM) [21]. The guide was structured around four overarching themes (perceptions of current services, desires for additional services, facilitators, and barriers), and questions/prompts relating to PRISM domains (characteristics of patient recipients, characteristics of organizational recipients, patient perspectives about the program, organizational perspectives about the program, external environment, and implementation and sustainability infrastructure) were interspersed throughout to ensure multi-level contextual factors were thoroughly explored [22]. The interview guide was designed in English then translated into Kiswahili. The interview guide was piloted with members of the research team, all Kenyan women fluent in English and Kiswahili and familiar with HIV and PMTCT services. Feedback from this process was used to refine the guide for clarity, flow, and contextual appropriateness prior to data collection.

After obtaining written informed consent, one of two research assistants utilized the interview guide to conduct one-on-one interviews with MMs and WLHIV. Both research assistants were Kenyan women with prior qualitative research experience, health-related degrees, and spoken and written fluency in English and Kiswahili. Their shared cultural and linguistic background, as well as familiarity with HIV and PMTCT services, may have facilitated rapport and openness among participants. At the same time, their professional roles as researchers may have influenced how questions were posed and how participants chose to respond. MMs were interviewed at their respective health facilities, outside of work hours. WLHIV were interviewed at the conclusion of routinely scheduled clinic visits, or at another mutually agreeable time and place. Interviews were conducted in English and/or Kiswahili, depending on participant preference. A brief sociodemographic survey was administered at the beginning of each interview. Each interview lasted approximately 60 minutes, and all participating MM and WLHIV were compensated 1000 Kenyan Shillings (approximately $10 US dollars) each for their time and effort. Interviews were audio recorded then translated (into English when obtained in Kiswahili) and transcribed verbatim by a trained translator/transcriber contracting with the AMPATH Qualitative Research Core (AQRC). No identifying details were included in the audio recordings or the transcripts. For quality assurance, one of the Kenyan investigators (MAK) periodically compared random sections of audio with their corresponding transcriptions to ensure integrity.

### Analysis

Theme-based content analysis was performed using NVivo 20 software (Lumivero, LLC; Denver, Colorado, USA). Two investigators (JGC and VN), in partnership with a trained coder contracted with AQRC, established an *a priori* coding framework corresponding to the study objectives and PRISM domains. Then, using the constant comparative method [23], existing codes were inductively refined and new codes were added as they emerged from the data. Coding was conducted iteratively, and discrepancies in coding were resolved by study team consensus. A thematic matrix generated by AQRC was used to organize codes and synthesize themes across interviews. Illustrative quotes were selected to highlight key themes. Data saturation was defined as the point at which no new codes or themes emerged during iterative analysis, and this was achieved by the completion of the 52 interviews, so no additional or repeat interviews were performed.

This manuscript was prepared following Consolidated Criteria for Reporting Qualitative Research (COREQ) guidelines [24] (S2 File).

### Inclusivity in global research

Additional information regarding the ethical, cultural, and scientific considerations specific to inclusivity in global research is included in the Supporting Information (S1 File).

## Results

### Participant characteristics

All 16 MMs at the three sites agreed to participate in the study. Their median age was 40 years (interquartile rage [IQR] 35–42 years), and they had been working as MMs for a median of 6 years (IQR 5–8 years). Seven (44%) of the MMs reported experience working in healthcare prior to becoming a MM, three of whom provided community-based services in their prior role. Two (13%) MMs had only primary education, seven (44%) had secondary education, and seven (44%) had post-secondary education.

Only one (3%) of 37 WLHIV approached declined participation in the study, citing that she was too busy. Among the 36 WLHIV who participated, median age was 29 years (IQR 26–35 years). Three (8%) WLHIV had no formal education, 21 (58%) had primary education, six (17%) had secondary education, and six (17%) had post-secondary education. Seventeen (47%) WLHIV reported living in a peri-urban area, 13 (36%) reported living in a rural area, and six (17%) reported living in an urban setting. Only one (3%) WLHIV reported not having a phone, 30 (83%) reported having their own phone, and five (14%) shared a phone with another person in their home or community. Median number of children per WLHIV was 2 (IQR 2–3). At the time of the evaluation, 33 (92%) WLHIV reported that their youngest child was less than 2 years old. Median household size (including the WLHIV, her children, and other adults) was 4 (IQR 4–6). Fifteen (42%) WLHIV reported having disclosed their HIV status to all other adults in their household. Twenty-nine (81%) WLHIV reported having a male partner, and 28 (97%) reported they had disclosed their HIV status to the male partner. By design, half of WLHIV were new positives and half were known positives. Among the known positives, three (17%) had been diagnosed with HIV prior to their most recent pregnancy but within the past two years, while 15 (83%) had been diagnosed with HIV more than two years prior.

### Perceptions of current Mentor Mother services

#### Mentor Mothers’ experiences with their clients.

MMs reported providing a variety of services for their clients, including: health education for antenatal and postnatal WLHIV and their infants; maternal ART and infant prophylaxis adherence counselling; facilitation of support groups; and, reminders for clinic appointments, maternal HIV viral load monitoring, and infant HIV diagnostic testing. Most MMs emphasized the importance of establishing a patient-centered, empathetic relationship with their clients. MMs also reported leveraging their lived experiences to build trust and address individual client needs; however, only one MM at Facility B explicitly reported prioritizing clients based on their level of need, stating, *“When they come, every client has her own issues… You will concentrate more to that client [with issues] than that client who doesn’t have any issue.”* Additional quotes illustrating MMs’ experiences with their clients can be found in Table 2.

**Table 2:** Mentor Mothers’ (MMs) experiences with their client women living with HIV (WLHIV).

Theme	Selected illustrative quote(s)
MMs have a strong, personal desire to serve other WLHIV	*“I've always had a desire of working in such a facility. Like there are situations when I would come to pick my drugs because I'm also HIV-positive. So, when I would come to pick my drugs, I would just imagine myself working here and talking to mothers, sharing my story with them… It's also something that I was longing for. So, being given that opportunity, It's amazing to me.”* ~ MM at Facility B
MMs establish patient-centered, empathetic relationships with their clients	*“I know them very well… we interact with them until they become our family. So we know everything about them.”* ~ MM at Facility A *“We love [our clients] because we are many and we are all in the same shoes, and whatever they are going through, I do too… I can relate.”* ~ MM at Facility C
MMs leverage their lived experiences to build trust and address client needs	*“I share about myself, how I overcome, because it is not very easy the first day when you are tested. You see as the earth has just swallowed you, it is not easy to recover. But when you find somebody who has gone through and encourage you, you will pick up.”* ~ MM at Facility A *“My interaction with my clients… has been good because most of the clients feel like I am more like them, so it’s like I understand them.”* ~ MM at Facility B

#### Clients’ experiences with Mentor Mothers.

Client WLHIV echoed MMs when describing the maternal-infant health education, adherence counselling, and retention support services provided to them through the MM program. Additionally, WLHIV reported that MMs provided personal advice about understanding and accepting a new HIV diagnosis, managing stigma and feelings of isolation, HIV-status disclosure, engaging male partners, family planning, intimate partner violence, and perinatal mental health problems. This advice from MMs was especially well received by WLHIV when grounded in shared lived experiences. WLHIV added that the kind and respectful interactions they have with MMs further promotes a therapeutic relationship and retention in care. This relationship was contrasted with the perceived mistreatment WLHIV sometimes received from other clinic staff. WLHIV also appreciated the deep and long-lasting relationships made with MMs, and they cited that empathetic reassurance and sharing of experiences allowed for open communication and instilled in them a sense of hope and vitality. A WLHIV at Facility A said, *“I had not yet healed, as in I was feeling like ‘why am I even living in this state, what am I doing living, what is this problem I brought upon my baby?’... But I feel like [the Mentor Mothers] have really helped me, because since that time till now I feel I’m released.”* Some WLHIV also mentioned occasionally receiving material support from MMs, including transport money and food, a practice the MMs saw as sometimes necessary in the short-term but not sustainable in the long-term. A MM at Facility A reported, *“Some [clients] come with expectations. They feel as if you are supposed to give them some money.”* Additional quotes illustrating clients’ experiences with MMs can be found in Table 3.

**Table 3:** Client women living with HIV (WLHIV) experiences with Mentor Mothers (MMs).

Theme	Selected illustrative quote(s)
Clients relate to and bond with MMs	*“It is the issue of the disease that has made us to be friends.”* ~ WLHIV at Facility A *“[My relationship with the Mentor Mothers] is important to me and I love them very much because they have taken care of me like their child.”* ~ WLHIV at Facility C
Clients value and are relieved by MM’s advice that is grounded in shared lived experiences	*“For me, the first time I knew my status, the manner in which they received me… they sat me down because it was shocking. So they had to talk to me, they bring me close, some [Mentor Mother] told me that even them it’s something they have gone through and so it is not the end of the world. So I was really happy with that kind of heart that they had. They encouraged me because I was in tears. How am I going to tell my husband? Then my husband came and got tested and was found negative, so I remained there asking myself how will it be in this house, will this marriage continue? Already I’m pregnant, in the house have three [children]. I felt like, What?!. But I found one [Mentor Mother], she guided me.”* ~ WLHIV at Facility B
Clients appreciate the respectful interactions they have with MMs	*“Even though I am sick they respect me. When they meet me with my baby, they are respectful. They don’t discriminate, like if you go to other places, you will find people look down upon you.”* ~ WLHIV at Facility C
Clients’ deep and trusting relationships with MMs facilitate open communication	*“You can share everything with them… I have never heard them discussing anyone… It is just between you two, just that.”* ~ WLHIV at Facility A *“Even if it is not the day for me to come to the facility to pick medicine, but I am just at home and feel like I miss them, I can pick up my phone and call them. We talk, they ask about my well-being, I ask about theirs, and life continues.”* ~ WLHIV at Facility C

#### Mentor Mothers’ experiences with other clinic staff.

The strong bond and deep understanding between MMs and their clients empowers MMs to advocate for them and, when needed, intercede with clinic staff on their behalf. This is consistent with MMs’ overall reporting of a collaborative work environment based on mutual respect and teamwork with other clinic staff. In particular, MMs reported collaborative efforts for defaulter tracing and caring for those newly diagnosed with HIV. MMs also reported benefiting from and feeling supported by knowledge sharing from other clinic staff. However, while some MMs felt that clinic staff recognized and valued MMs’ unique perspective as both patients and providers, other MMs cited instances when clinic staff stigmatized MMs and WLHIV because of their HIV status. Selected quotes illustrating MMs’ experiences with other clinic staff can be found in Table 4.

**Table 4:** Mentor Mothers’ (MMs) experiences with other clinic staff.

Theme	Selected illustrative quote(s)
MMs work collaboratively with other clinic staff, advocating on behalf of their clients when necessary	*“Maybe there are some issues that the client has told me, especially the new clients, she may feel too overwhelmed to share with the clinician. So I also get to work with clinician.”* ~ MM at Facility A *“Someone may even go to the doctor, talk, and finish, but later when she is leaving you will hear her say that ‘I had a problem but I was uncomfortable sharing.’ So, she will reframe to me the problem so that I again take it back to the doctor.”* ~ MM at Facility B
MMs appreciate being supported by and learning from other clinic staff	*“I get to learn new things every day. [The other clinic staff] are also helpful… they teach me how to operate [the electronic medical record], how to interact with the [clients], what I should do. Yeah, so I would say the relationship is actually great.”* ~ MM at Facility B
MMs sometimes experienced stigmatizing attitudes from other clinic staff	*“There are some nurses who stigmatize others. Even just by hearing the word virus when you are talking ‘aah-aah’... Nurses who haven’t studied such things, shouldn’t care for these clients... Talk to her well... When she gets bad service she will never come back.”* ~ MM at Facility B

#### Perceptions of the Mentor Mother program structure, roles, and responsibilities.

MMs typically meet their client WLHIV at the health facility during approximately monthly antenatal and postnatal visits. Occasionally, MM-client interactions are more frequent if a WLHIV has active issues (e.g., comorbid maternal or infant medical conditions, pregnancy complications, psychosocial problems, etc.), and less commonly clinic visits are spaced out to every three months for select stable clients (e.g., postpartum WLHIV with sustained HIV viral suppression and no other active issues). Either way, the frequency of clinic visits is typically driven by the medical team rather than a MM’s assessment of client needs.

In some cases, WLHIV might have continuity with the same MM at every encounter, but generally WLHIV meet with whichever MM is available on any given day. MMs typically meet one-on-one with WLHIV in a dedicated room at the health facility, but due to crowding and thin partitions privacy is not always guaranteed. According to a WLHIV at Facility C, *“You can talk but I don’t feel it is that private because the other side has patients, so you have to talk in low tones.”*

Between visits, MMs might contact patients for various reasons (e.g., appointment reminders, follow-up for missed appointments, or to offer support during times of distress). Likewise, a WLHIV might reach out to a MM between visits if they have a new concern or to seek ongoing support for a persistent issue. When interacting between scheduled visits, both MMs and WLHIV generally preferred phone calls rather than text messages because of privacy concerns; though, this depended on whether the WLHIV shared a phone with other household members, and it was less of a concern when WLHIV had disclosed their HIV status to others in the household. A MM at Facility A stated, *“I always call; I don't like sending messages. Mostly I call and talk, because if you send some messages, most of the clients sometimes they use their phone with others and they have not disclosed [their HIV status].”*

MMs and their client WLHIV also appreciated opportunities for home visits; though, these are not routine in the current program structure. As with phone calls and text messages, home visits are limited by privacy and disclosure concerns, and capacity for home visits is further constrained by limited staff and resources for outreach. The main scenario where MMs can make home visits is when tracing clients who have been lost to follow-up; however, MMs do not currently have the discretion to perform home visits based on perceived client needs.

When asked how their perceptions of the MM program have changed over time, some MMs reported frustration about their role evolving from a focus on mentorship and counselling to a broader set of duties they perceived to be outside of their primary role. They worried that these additional tasks interfere with their ability to adequately serve their clients. MMs also expressed dissatisfaction with decreases in compensation despite increased expectations and rising costs of living. A MM from Facility C stated, *“Previously, the Mentor Mothers used to be paid through the bank, then it changed to stipend, and they even lowered the salary not like before... the cost of living is going up and keeps going up.”* Compounding these issues, MMs reported lack of a clear reporting structure, with one MM from Facility B stating, *“When we have a problem, we don’t know whom we are to report to.”* Client WLHIV, on the other hand, generally felt as though the MM program has remained consistent over time. WLHIV did not seem to perceive the frustration experienced by the MMs; rather, their appreciation for MMs increased as they developed relationships while navigating through PMTCT services.

In terms of MMs’ ability to identify and discuss issues that are personally relevant to client WLHIV, MMs and WLHIV reported some success with addressing various medical, psychosocial, and economic needs. WLHIV generally felt comfortable bringing up such issues with MMs and were satisfied with MMs’ support related to these issues. MMs shared a sense of satisfaction when able to identify and address WLHIV’s needs; however, MMs also felt limited in their ability to provide support and services for many of these issues within the current program structure. Specifically, MMs are not currently empowered to risk stratify their clients, nor are they provided with the targeted training and resources that would facilitate adequately addressing these and other issues.

Additional quotes illustrating MMs’ and WLHIV’s perceptions of current MM services can be found in Table 5.

**Table 5:** Mentor Mothers’ (MMs) and client women living with HIV (WLHIV) perceptions of current MM services.

Theme	Selected illustrative quote(s)
MMs derive joy from addressing clients’ needs	*“When you have addressed a client's problem satisfactorily you go home feeling happy. As the client came unaware of the options and she did not know if she would be able to get assistance but came in and helped.”* ~ MM at Facility A
The frequency of clinic visits is typically driven by the medical team, but scheduling could be more responsive to MMs’ assessment of client needs	*“We will see the mothers according to their needs. If you are in antenatal care, you have high viral load, you are a newly tested [positive for HIV], we shall see you frequently… If you have [undetectable viral load], you are a known positive… those mothers we have walked the journey, they know, we have carried the first pregnancy with them, the second… we don’t see them frequently.”* ~ MM from Facility A *“When you tell [the clinician] that this mother is stable, she can come maybe after two months, they refuse saying that that mother must be seen every month, and that is the rule.”* ~ MM at Facility B
MMs reported frustration with being tasked with duties that they perceived to be outside of their scope of work	*“Currently, at times we feel as if we are misused. At times you are the patient attendant, at times you are the pharmacist, at times you are sent to bring medicine. We are basically being overused. Can be very many compared to the duties that you are supposed to do. That is not fair.”* ~ MM at Facility A *“If you were taught to work in a certain way and you find that you can no longer work that way anymore, you become demoralized.”* ~ MM at Facility B
MMs and their clients appreciate home visits, but these are not routine and have to be balanced against privacy concerns	*“If you are keeping something private, neighbors will start asking ‘why?’”* ~ WLHIV at Facility A *“At some point there is money that we are given to go and pay the client a visit at their home, if the client cannot be reached on the phone and the virus level is high, at least we go to the house and also see how does she store these drugs, how is the house like.”* ~ MM at Facility B
WLHIV were generally satisfied with current MM services	*“The benefit that I have received, is very big for sure. I’m very grateful for the teachings as well. I’m grateful for their volunteering to help, I’m very grateful.”* ~ WLHIV at Facility B *“[Mentor Mothers] has increased its tenderness because they are always close to me, they constantly ask about my wellbeing. I feel like currently the built friendship has been heightened and they have become like sisters.”* ~ WLHIV at Facility C
MMs are accessible, and clients appreciate being contacted both during and in between scheduled visits	*“These [Mentor Mothers] are always at the clinic. You come anytime, any day, you will find them. Even if you have some problem, if you just run to them you will find them and they will help you.”* ~ WLHIV at Facility B *“I like it when they call us, when they visit us all the time, when they visit the sick.”* ~ WLHIV at Facility C
MMs have limited time, training, and resources to identify and address client needs	*“When they come to the clinic, we do not have time to explore to talk about everything.”* ~ MM at Facility A *“When there is an opportunity of going for a course, other people are given such opportunities and not Mentor Mother, yet you are the one who struggle with the patients.”* ~ MM at Facility B *“Additional resources are needed… resources that can enable us to support the client and the Mentor Mothers will be good.”* ~ MM at Facility C

### Desired modifications to the Mentor Mother strategy

Beyond the maternal-infant health education, adherence counselling, and retention support services routinely provided through the MM program, MMs and client WLHIV identified a similar list of medical, psychosocial, and economic issues that are commonly encountered by WLHIV as they navigate PMTCT services and that could potentially be identified and addressed through peer support. MMs expressed a strong desire for additional training and resources to help them identify and meaningfully address these issues. MMs were also interested in having deeper engagement in medical case management as part of multidisciplinary care teams that already exist for clients with non-adherence to ART and/or HIV viremia, increased collaboration with community health workers for provision of decentralized services, and the possibility of integrating economic empowerment activities into support groups.

When specifically asked what they thought about the idea of modifying the MM program such that some services are allocated based on a client’s specific needs, some MMs and client WLHIV were hesitant, but most were agreeable and even excited about the possibility of risk-stratified differentiated service delivery. A WLHIV at Facility A said, *“I would like that so much. Let the clients who are coming in be given the services that they need, and the other person as well goes to get their services, not waiting for this one to finish.”* MMs proposed that many of the issues commonly encountered by WLHIV as they navigate PMTCT services could serve as the basis for risk stratification and problem-focused services. For example, a MM from Facility A said, *“It is possible, because when you encounter a mother you are the first person who encounter. We look at that mother and see this mother has some needs, this mother is well off.”* Likewise, MM and WLHIV were interested in avoiding redundant or unnecessary services for WLHIV who do not have identifiable issues. MM more specifically supported the idea of reducing the amount of time they spend with stable clients so that they can spend more of their time and resources to assist clients with identifiable needs.

MM and WLHIV also expressed a shared desire for more decentralized services, potentially including home visits or meeting in the community for preventative or problem-focused support. Preferences for decentralized services were also at least in part motivated by a desire to reduce the burden of frequent, usually monthly, client travel to and from clinic. Though, it was acknowledged by both MM and client WLHIV that community-based encounters would need to be balanced against pervasive HIV-related stigma in the community, and that being the case any decentralized services would need to be carried out discretely and only after receiving permission from WLHIV. A MM from Facility B reported, *“If they are seen with [a Mentor Mother in uniform]… and maybe she is with her neighbor, she will disappear.”*

MMs and client WLHIV also saw value in promoting more male partner involvement; however, there was less certainty about how to consistently and successfully engage male partners in the setting of non-disclosure of a WLHIV’s HIV status and in the face of prevailing gender roles in this setting. A WLHIV at Facility B shared, *“They are scared to tell their husbands, and that maybe their husband can decide to leave them because they are on medication.”*

MMs expressed dissatisfaction with their current level of compensation, but in addition to desiring better pay they were also interested in receiving employer benefits like health insurance, more appreciation from other clinic staff, and possibly formal certification for their work. Interestingly, some client WLHIV also had insight into the plight of MMs, with one WLHIV from Facility B commiserating, *“In connection with the Mentor Mother what can make them tired is if the government will not stand with them. They should also get to be motivated because you see, there are some jobs that you can do but then if you are not paid well, you get tired. And if they ever get tired, it is us who will suffer.”*

Both MMs and client WLHIV also desired additional resources to address the material needs of WLHIV and their babies. Engagement of WLHIV is further constrained by healthcare facility fees, with a MM from Facility A reporting, *“Many clients are crying about the amount to be paid.”* Beyond direct provision of resources and advocating for lower fees, MMs also suggested that linking WLHIV to income generating activities could be a cross-cutting strategy to promote economic empowerment and as well as HIV-related health outcomes. Client WLHIV were also supportive of and eager to engage in economic empowerment activities.

Additional quotes illustrating MMs’ and WLHIV’s desired modifications to the MM strategy can be found in Table 6.

**Table 6:** Mentor Mothers’ (MMs) and client women living with HIV (WLHIV) desired modifications to the MM strategy.

Theme	Selected illustrative quote(s)
MMs desire better pay, benefits, appreciation, and possibly formal certification for their work	*“First of all, I could recommend the Mentor Mothers to be appreciated… Mentor Mothers should be taken to a training and given Mentor Mother membership certificate.”* ~ MM at Facility A *“What I do not like at all is the way the program itself pays the Mentor Mothers, because what a Mentor Mother does, if you compare with the income of the work she has done, they do not get along.”* ~ MM at Facility C
MMs and client WLHIV expressed frustration about long wait times at the clinic	*“Mothers see that we are wasting her time.”* ~ MM at Facility A *“When you come here, someone can decide to come very early from home, maybe by eight hoping that by nine you are home. But then now you find that you come and take so long. You come at eight, you stay here till eleven, twelve, that is when you are leaving. So what I would like to recommend is there about time.”* ~ WLHIV at Facility B
MMs and client WLHIV understood and were generally agreeable to the idea of risk-stratified differentiated service delivery	*“I would put more time, more resources, more efforts to this one who has a need as opposed this one. I would give both the service, but I would put more emphasis on this one who has special needs.”* ~ MM at Facility A *“There is no need of bringing some clients and making them queue. Better that idea, so that the one who really needs service can have enough time.”* ~ MM at Facility B *“For example, someone who is suppressed, this person's service will be short term and does not take long but when someone is unsuppressed it takes a long time.”* ~ MM at Facility C
MMs and client WLHIV desire more community engagement and home visits	*“Maybe because of one reason or another she doesn’t want to come here, and she is requesting that we see her at home… And if we can reach her, we can take to her the services wherever she is instead of her coming… We do all those things wherever she is instead of her coming to seek services.”* ~ MM at facility B *“There are people in the interiors who are hiding and they don’t accept it… [The Mentor Mothers] should walk around and tell those others, there in the rural, let them tell them that having HIV is not the end of life you can still continue, you can be able to bring up your children.”* ~ WLHIV at Facility A *“During antenatal, women most of the time go through a lot of challenges… So I would like if they can be coming to visit the pregnant mothers and at least encourage them. You know at times you might be carrying a pregnancy but are stressed, so I would like if they came… I would like for these Mentor Mothers to have additional time to come and follow up on these mothers while in the community.”* ~ WLHIV at Facility C
MMs and client WLHIV desire additional resources to address the material needs of WLHIV and their babies	*“There are those mothers who refuse to take their drugs because they don’t have food. If it was possible to offer them things like flour, it could be better. And, also the issue of transport, you find that some refuse to come to the clinic because they do not have transport.”* ~ MM at Facility B *“So the facility or organization can form a program like for every postnatal visit she is given something small, and also go with milestones, for example when a child has reached 6 months you are given healthy foods for the child and mother.”* ~ WLHIV at Facility C
MMs and WLHIV desire income-generating activities to promote economic empowerment and better HIV-related outcomes	*“Economic empowerment is also an additional service that these clients can benefit from. Most of our clients are socio-economic. By virtue of being socio-economic affects the way they come to the clinic, retention, adherence… It can really help.”* ~ MM at Facility A *“I would like to receive from them… something like business you see. At least let us be talking about issues like now about business so that at least for someone who wants to start something like their own business, she can know where to get support.” ~ WLHIV at Facility A*
MMs and client WLHIV saw value in and desire more male partner involvement	*“There is a client who has a husband and they are discordant... We called him saying she is sick and he turned up, that is when we started educating him about HIV... He doesn’t know anything, all he knows is that if someone has HIV, then she would infect him too. Up to date, they are still discordant and imagine it is the husband who brings her to every clinic visit.”* ~ MM at Facility B *“Male partners’ support and understanding can enhance a woman's adherence to treatment.”* ~ MM from Facility C
Clients desire support for assisted disclosure of HIV status to family and male partners	*“I have known my status and my partner does not. You see I will use those Mentor Mothers to plan so that my partner also can come so that we can get to know if he also has it, or maybe they will give me advice how I will go and tell him, as in so that it does not cause problems.”* ~ WLHIV at Facility A

### Barriers and facilitators to an enhanced Mentor Mother strategy

Despite both MMs and client WLHIV expressing many desired modifications to the current MM strategy, as well as generally being supportive of adapting the program to provide risk-stratified differentiated service delivery, several potential barriers to implementation were identified. Firstly, differentiated service delivery would require new workflows and additional MM tasks, which would need to be balanced against MMs’ desire for a clearer scope of work, less administrative tasks, and more time with clients in need of additional support. However, MMs perceived that if low-risk clients can fast track through the clinic and/or have less frequent clinic visits, this could enable MMs to spend more time providing services for high-risk clients, potentially without a net increase in MM workload. MMs also suggested that a clearer scope of work could potentially improve overall clinic efficiency, decreasing wait times and resource requirements.

The combination of generalized poverty amongst client WLHIV and health-system resource constraints also presents barriers to care. Some WLHIV mentioned occasionally receiving material support from MMs to help mitigate this barrier; however, MMs themselves have complained about insufficient compensation for their work and limited resources to adequately address client needs. Congruent with MMs’ dissatisfaction with their current level of compensation and recognition for their work, MMs similarly reported that increased support could help motivate and sustain their ability to assume additional responsibilities. Specifically, MM would appreciate better compensation, benefits, recognition, and possibly certification for their work. Without a lot of additional resources, small health system investments in MMs (e.g., providing health insurance) and resources for clients (e.g., providing food and transport assistance or reducing fees) could improve morale and address client and MM needs.

MMs cited instances when clinic staff stereotyped and stigmatized MMs because of their HIV status and relatively lower level of education compared to other clinic staff, and they worried that if not addressed this could pose challenges to implementing a strategy that empowers MMs to perform risk stratification and deliver problem-focused services for their clients. A MM at Facility B reported, *“Dealing with some of them is difficult. Actually, they always say ‘you’, especially when they see clients who are [HIV] positive. They refer to them as ‘these clients of yours’… They feel that Mentor Mothers’ work is for those who are not learned… So they look down upon us so much.”* However, MMs also reported that some clinic staff recognized, valued, and benefited from MMs’ unique positionality as both patients and providers. A MM from Facility A shared, “*Some mothers cannot open to the doctor. So when you know anything about the client, you quietly talk to the doctor in that when the client reaches there, he has the full picture of the mother.*”

Privacy concerns need to be addressed for MMs to effectively interact with client WLHIV and identify their individual needs, some of which are deeply personal. MMs reported that they attempt to meet with clients in a private space allowing for open and honest conversation; however, some WLHIV reported that interactions were sometimes in locations that were not conducive to private and open conversation. While both MM and client WLHIV desired stronger protections to ensure privacy and confidentiality in the clinic setting, changes to clinic infrastructure are probably not realistic within existing resource constraints; however, refresher trainings about the importance of confidentiality and implementing corrective measures when breaches in confidentially occur would not require much if any additional resources. Other creative approaches to promoting privacy and confidentiality were also suggested by MMs and client WLHIV, including avoiding text messages and providing more decentralized services. Though, as it pertains to community engagement and home visits, MMs should avoid wearing health worker or HIV program uniforms or logoed attire, since this was reported as a barrier to engagement.

The current lack of funds allocated for outreach activities is also a potential barrier to community engagement and home visits. A MM from Facility B shared, *“It reaches a time when there are [high-risk clients] who cannot be visited. Then there is a client that needs to be monitored but they say that they are unable to fund client to come. Then a client who cannot take the medicine no matter which tactics we use including deciding to supervise her to take the medicine from the facility. But now it reaches a time we don’t have that kind of support and so the facilitation lacks. So, we just sit here and wait for the client to come to the facility.”* To address this issue, MMs suggested that enhanced health facility and MM cooperation with community health workers could help address these clients’ needs, but they also recognized that additional training and sensitivity to privacy and stigma concerns would be essential for successful community engagement with WLHIV.

An overarching barrier that impacts all the aforementioned issues is pervasive stigma – internalized and anticipated stigma at the individual-level, and experienced stigma at the facility-, household-, and community-level. Participants described stigma as influencing male partner engagement and complicating disclosure of their HIV status to male partners. They also highlighted experience sharing, such as MMs disclosing their own HIV status to client WLHIV and participation in psychosocial support groups, as potentially helpful for coping with and addressing stigma. The deep connection and storytelling that occurs between MMs and client WLHIV, but not with most other clinic staff, can be therapeutic for clients learning to accept their HIV diagnosis and the routine of HIV care and treatment. Additionally, economic empowerment activities could enhance WLHIV’s self-esteem and decrease their dependence on male partners, potentially allaying internalized and anticipated stigma, as well as fear of mistreatment or abandonment from their partner.

Additional quotes illustrating potential barriers and facilitators to an enhanced MM strategy can be found in Table 7.

**Table 7:** Potential barriers and facilitators to an enhanced Mentor Mother (MM) strategy providing risk-stratified differentiated services for client women living with HIV (WLHIV).

Barriers	
Theme	Selected illustrative quote(s)
Pervasive HIV-related stigma in the community results in privacy concerns	*“When someone goes there wearing that ‘Kata shauri’ [slogan of a national PMTCT campaign], that is when you find that client will not want to see the Mentor Mother because they are wearing Kata shauri and this name AMPATH.”* ~ MM at Facility A *“Clients who have not disclosed their status may resist home visits due to privacy concerns.”* ~ MM at Facility B
Privacy/confidentiality concerns at the health facility	*“Space constraints and mixed clinics can result in discrimination and accidental disclosure of HIV status.”* ~ MM at Facility C *“Some fear coming to the facility because their neighbor works there or people from her area also attend that facility and she does not want to be seen where she is entering.”* ~ WLHIV at Facility C
Difficulty with engagement and disclosure of HIV status to male partners	*“They are scared to tell their husbands. And that maybe their husband can decide to leave them because they are on medication.”* ~ WLHIV at Facility B *“If my husband could come for testing… to get checked on his [HIV] status, so when they call him and he refuses.”* ~ WLHIV at Facility C
Poverty and lack of sustainable solutions to economic barriers to care	*“The challenge that we get is that a client comes to the facility when they have depleted the Linda Mama [government-funded initiative that covers some perinatal health services]. You find that someone says that they did not have transport... They do not have 150 [Kenyan Shillings; ~$1 US Dollar].”* ~ MM at Facility A *“These women are suffering. They don’t have transport, food and even soap. And, maybe, I have been given only my transport, but when you get there she has no transport for herself and the baby… So, I will have to bother my colleagues and sometimes they also don’t have money to spare for it to be possible to bring her to the clinic.”* ~ MM at Facility C
Linking/referring clients to specialty services is limited by availability and accessibility	*“The distance between various clinics is a barrier, and clients may not reach the intended referral point.”* ~ MM at Facility A *“Quality of service and long waiting times at referred clinics affect clients’ willingness to seek referrals.”* ~ MM at Facility B *“Lack of money for transport and hospital charges presents a barrier to accessing referred care.”* ~ MM at Facility C
Client migration across the Uganda border presents language barriers and challenges with patient tracing	*“Since we are at the border, sometimes language becomes a challenge… The Ugandan’s they usually speak Samia or Luganda… Very few usually speak English and we are used to Swahili, so communication is usually a challenge.”* ~ MM at Facility B *“You cannot cross over without informing Ugandan authority that you are going to look for such and such a client. We usually communicate to them… They want you to pay them some that they can help you trace them.”* ~ MM at Facility B
**Facilitators**	
**Theme**	**Selected illustrative quote(s)**
Building capacity for MMs to provide problem-focused services will be integral to a risk-stratified MM strategy	*“Adherence training and family planning training are needed for Mentor Mothers, as well as ongoing training to keep up with changing practices.”* ~ MM at Facility A *“You want this Mentor Mother to be able to do triage, you must just take her for proper training.”* ~ MM at Facility B *“Once a year or twice a year and it should be duration of 2 weeks, and refresher courses should be done quarterly.” ~ MM at Facility C*
A clearer scope of work could improve MM morale and efficiency	*“The scheme of work, improve on it so that it specifies what Mentor Mothers need to be actually doing. Then it should be structured well, and people should also be empowered or made aware about the work of the Mentor Mothers. I feel that this is lost.”* ~ MM at Facility B
Decentralized services could help mitigate stigma and financial barriers to care	*“If we could have community support groups it can be better, fight stigma, contact tracing will be very easy, we will have fought the stigma.”* ~ MM at Facility B *“In submitting antiretrovirals, they should be categorizing patients based on their locations. For example, people from one area should have their medicines picked from a particular Mentor Mother, whether it is from a predetermined venue or given to their homes in their same community so as to reduce transport expenses, because there are some who are persistently in need of transport and this will reduce the rate of defaulters… When they bring the medicine they can have a small discussion and a checkup of their progress, rather than just dropping the medicine and leaving.”* ~ WLHIV at Facility C
Cooperation with community health workers	*“Like for the mothers who live far, there are [community health workers] who also come from far places and can come and pick the medicines and take to them.”* ~ MM at Facility C
Stigma reduction efforts and self-acceptance can empower WLHIV to achieve and promote better health outcomes	*“The first thing you need to do is accept yourself. It becomes problematic if you have not accepted yourself, but once you have accepted yourself it gives you the courage that even if you were to stand before 1,000 people you can express yourself with courage because with that you will be helping the one who is still closed to come out. Personally, I am not so shy as to hide my status. I disclose it and even explain how I got infected so that those who are unaware of their status can come out and get tested so that she can start treatment.”* ~ WLHIV at Facility C
Opportunities for experience sharing are effective at reducing stigma and feelings of isolation and despair	*“When someone is identified to be positive you are always stressed, you get confused, you even wish to be dead. But now if they are there, they can talk to you, and then the good thing, even her herself will tell you, ‘Even me I am positive’. So you get to be fine.”* ~ WLHIV at Facility A *“I would like that they continue being the way they are, receiving someone, guiding them and to accept them. That make someone accept herself and you feel like you are not… that loneliness disappears from you because of those [Mentor Mothers] of ours.”* ~ WLHIV at Facility B
Motivating MM and WLHIV through appreciation and material support could boost morale by improving job satisfaction and security	*“The incentive would be to put them on payroll. Currently no Mentor Mother, all of them are working on voluntary basis, which is not job. We do not have job security yet you are trying to solve a client’s issue so that she can get a start-up kitty yet you are not able to benefit from it.”* ~ MM at Facility A *“We are ready for change. In fact, we want that change, we want to be recognized. That is the thing, we have done, we have worked like ‘volunteerish’ for long years, for many years, so we want to be recognized.”* ~ MM at Facility B *“If possible, they should Mentor Mothers’ payments through a pay slip system, providing for us [health insurance] cover will make the program better.”* ~ MM at Facility C

**Fig 1:**
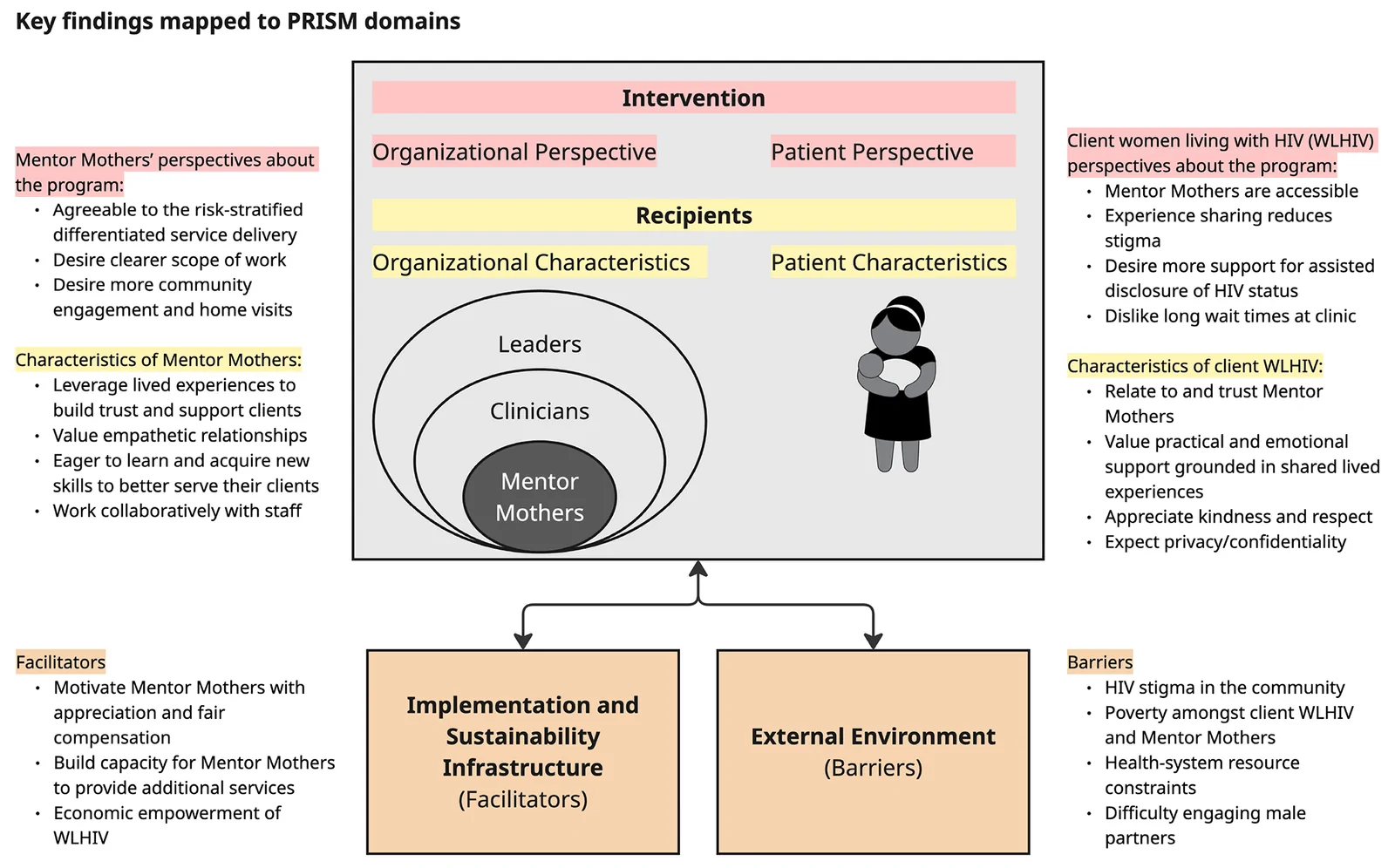
Summary of key findings mapped to Practical, Robust Implementation and Sustainability Model (PRISM) domains. Adapted from: Feldstein AC, Glasgow RE. *Jt Comm J Qual Patient Saf. 2008 Apr;34*(4)*:228-43.*

Key findings are mapped to PRISM domains in Fig 1.

## Discussion

In-depth qualitative interviews with MMs and their client WLHIV at three health facilities providing PMTCT services in western Kenya revealed important insights about the existing MM program, as well as perspectives on how to enhance the MM program such that it provides more patient-centered, individualized services to promote improved maternal-child health outcomes and quality of life. Key findings included that both MMs and client WLHIV had favorable opinions about the possibility of developing and implementing a risk-stratified MM strategy wherein client WLHIV with identifiable risk factors receive intensified problem-focused services while low-risk WLHIV are eligible for less intensive services. MMs and client WLHIV also agreed that empathetic MM-client relationships that leverage shared lived experiences to build trust and reduce stigma should remain central to the MM program. While some MMs reported experiencing stigma from clinic staff, which could hinder implementation of expanded MM roles, others reported that staff valued MMs’ unique perspectives as both patients and providers. Therefore, with additional MM training and recognition of newly acquired skills, staff culture could evolve to be more supportive, especially if demonstrating impact on important clinical outcomes. However, MMs were clear that a better-defined scope of work and capacity building through training and skills development would be necessary for successful implementation of an enhanced MM strategy. Additionally, motivating MMs with fair compensation and recognition for their work, as well as providing more resources for client WLHIV with economic barriers to care, could promote adoption and sustainability of an enhanced MM strategy.

Differentiated service delivery models for HIV care and treatment have been promoted by the World Health Organization and the Kenya MoH for about a decade [25–27]. However, only in recent years have guidelines endorsed differentiated service delivery for WLHIV who are engaged in PMTCT services [28–30]. The primary purpose of these differentiated service delivery models has been to increase clinic/program efficiency by streamlining services for stable clients, and in practice this has translated into WLHIV who are receiving ART and who have sustained viral suppression being eligible for less frequent clinic visits and multi-month dispensing of ART (e.g., every 3–6 months, rather than monthly). In Kenya, MMs and WLHIV have expressed preferences for this approach [12,31,32], and there is recent evidence that clinic visit spacing for stable PMTCT clients has been increasing annually since 2019 and has resulted in improved retention in care [33]. While this approach addresses the preferences of stable low-risk WLHIV, it does not leverage the rich lived experiences of MMs to address the needs of high-risk PMTCT clients. That said, this study is amongst the first to explore the idea of utilizing MMs to provide more intensive services for PMTCT clients with identifiable risk factors, rather than just focusing on less intensive services for low-risk clients. Our findings are novel in demonstrating how a risk-stratified MM approach, prioritizing intensified support for higher-risk clients, emerges alongside important considerations about MM role clarity and workload, which may shape the feasibility and scalability of such models. Our findings extend prior work on differentiated PMTCT service delivery by shifting the focus from streamlining care for stable clients to leveraging MMs to deliver intensified, individualized support for higher-risk WLHIV. It is also worth noting that while MMs are not formally recognized or certified to provide services outside of their current scope of work, they are already informally and without much support leveraging their rich lived experiences to serve their clients to the best of their abilities.

By offering low-risk clients less intensive support, MMs should have more time and resources to devote to clients with identifiable risk factors (e.g., HIV viremia, non-adherence to ART, missed clinic visits, stigma, non-disclosure of HIV status, perinatal mental health issues, and other socioeconomic barriers to care). Therefore, on balance, a risk-stratified MM strategy might not require any additional resources, and might even enhance clinic efficiency, patient outcomes, and quality of life. Adjunctive services for high-risk client WLHIV, such as increased community engagement, home visits, economic empowerment activities, assisted disclosure of HIV status, and male partner engagement, could be layered into the package MM services provided to clients based on their individual needs. In fact, there is already evidence that MMs and other lay health workers are capable of effectively providing some of these services if properly trained and capacitated to do so. For example, in the Mother and Infant Retention for Health (MIR4Health) clinical trial in Kenya, a multi-component MM-administered intervention that included individualized education and counselling resulted in lower mother-infant attrition at 6-months of follow-up [7]; though, the relative efficiency and cost of administering the complex package of services to all WLHIV regardless of risk-attributes was not addressed in this study. MMs found the MIR4Health intervention to be feasible and acceptable, and focus group discussions revealed that MMs administering the MIR4Health intervention played an important role in supporting WLHIV across a range of behavioral, social, and structural domains, while also identifying potential barriers to uptake that mirrored those identified in our assessments of MMs and client WLHIV – privacy and stigma concerns, practical limitations of the healthcare system, and healthcare worker attitudes [2]. In the per-protocol analysis of Mother-Infant Visit Adherence and Treatment Engagement (MOTIVATE) clinical trial in Kenya, community-based MMs performing structured home visits (compared to standard of care facility-based MMs) resulted in improved 12-month retention in care [8]. MOTIVATE also showed that stigma, depression, and domestic violence further modulate PMTCT outcomes, indicating a need to develop tailored MM strategies that can respond to the specific needs of mother-infant dyads [11]. For example, training modules focused on stigma reduction, supported HIV-status disclosure, male partner engagement strategies, and basic perinatal mental health screening and counseling could be particularly impactful and consistent with MMs’ desire for additional training as well as the needs identified by both MMs and client WLHIV.

In the absence of clear evidence about the relative impact of the various services that could be provided by MMs, contextualized prioritization of intervention components based local perspectives about feasibility and potential impact should be considered. For example, while MMs and client WLHIV in our study expressed strong desires for more community engagement and male partner involvement, pervasive stigma and patriarchy in the western Kenya context pose major roadblocks. At Facility B, where cross-border client migration with Uganda was common, additional challenges related to language barriers and patient tracing further underscore the need to tailor implementation strategies to local context. On the other hand, there has been great success with integrating economic empowerment activities into community health worker-led, group-based maternal-child health promotion activities in western Kenya [34–37], so prioritizing this component for initial piloting might be more acceptable, feasible, and appropriate. This approach could also serve as the scaffolding for future implementation of additional high-risk pathways that were not prioritized for initial implementation. For example, while MMs are not currently capacitated to address perinatal mental health problems, in the future they could be trained in mental health interventions (e.g., problem-solving therapy or motivational interviewing) that have been successfully implemented for use by lay health workers in similar contexts [38–45].

The main strength of this study was our rigorous, in-depth evaluation of both individual (i.e., client WLHIV) and organizational (i.e., MM) perspectives about MM services, guided by the PRISM model. Purposive sampling of individuals from distinct facility types, stratified by recency of HIV diagnosis and PMTCT outcomes, also helped ensure representation of diverse perspectives. Research assistants were trained to use the PRISM-informed interview guide to ask open-ended, non-leading questions; however, since we approached this evaluation with the objective of gathering formative information to guide the development of an enhanced MM strategy, it is possible that social desirability bias could have influenced some participants’ responses. Another limitation is that some issues like poverty and stigma are pervasive and external to the MM program, so additional participatory research gathering input from system-level stakeholders is needed to ensure that feasible enhancements to MM services are contextualized within the real-world constraints of PMTCT programs. Additionally, while we expect that our findings will be generalizable to the western Kenya context, additional research is needed to determine whether our findings will be readily transferable to other settings in Kenya, or sub-Saharan Africa in general, particularly outside of AMPATH-supported clinics where program structure and resources may differ.

## Conclusions

This qualitative evaluation of MMs and their client WLHIV in western Kenya highlights strong support for an enhanced, risk‑stratified MM strategy that allocates more intensive, problem‑focused services to clients with identifiable risk factors while streamlining support for low‑risk clients. Participants emphasized that empathetic, trust‑building relationships rooted in shared lived experiences should remain central to MM services, and that successful implementation will require a clear scope of work, additional training, and fair compensation for MMs. A risk-stratified MM strategy has the potential to improve program efficiency, maternal-child health outcomes, and quality of life by freeing time and resources for MMs to provide high‑risk clients with individualized services such as economic empowerment, assisted disclosure, and community engagement. Prioritizing intervention components that are acceptable and feasible within the local context may offer a practical starting point for piloting, with opportunities to expand to additional high‑risk pathways over time. Continued participatory work with system‑level stakeholders will be essential to ensure that enhancements to MM services are responsive to barriers, including stigma and poverty, that extend beyond the MM program.

## Supporting information

S1 Text In-depth interview guide used for data collection in this study, with questions and optional prompts for Mentor Mothers and postpartum women living with HIV who have been served by Mentor Mothers. (DOCX)

S1 File PLOS Inclusivity Questionnaire: Author responses to the PLOS Inclusivity in Research Questionnaire which explores ethical, cultural, and scientific considerations specific to inclusivity in global research. (DOCX)

S2 File COREQ Checklist: Consolidated criteria for reporting qualitative research checklist items and where the corresponding section can be found in this manuscript. (DOCX)

## References

[1] Joint United Nations Programme on HIV/AIDS U. UNAIDS Data 2024. Geneva, Switzerland: Joint United Nations Programme on HIV/AIDS. 2024. https://www.unaids.org/en/resources/documents/2024/2024_unaids_data

[2] DiCarlo A, Fayorsey R, Syengo M, Chege D, Sirengo M, Reidy W, et al. Lay health worker experiences administering a multi-level combination intervention to improve PMTCT retention. BMC Health Serv Res. 2018;18(1):17. doi: 10.1186/s12913-017-2825-8 29321026 PMC5763814

[3] Onono M, Odwar T, Wahome S, Helova A, Bukusi EA, Hampanda K, et al. Behavioral Interventions can Mitigate Adverse Pregnancy Outcomes Among Women Conceiving on ART and Those Initiated on ART During Pregnancy: Findings From the MOTIVATE Trial in Southwestern Kenya. J Acquir Immune Defic Syndr. 2021;86(1):46–55. doi: 10.1097/QAI.0000000000002521 33306563 PMC7851482

[4] Sam-Agudu NA, Ramadhani HO, Isah C, Anaba U, Erekaha S, Fan-Osuala C, et al. The Impact of Structured Mentor Mother Programs on 6-Month Postpartum Retention and Viral Suppression among HIV-Positive Women in Rural Nigeria: A Prospective Paired Cohort Study. J Acquir Immune Defic Syndr. 2017;75 Suppl 2:S173–81. doi: 10.1097/QAI.0000000000001346 28498187

[5] Cataldo F, Sam-Agudu NA, Phiri S, Shumba B, Cornelius LJ, Foster G. The Roles of Expert Mothers Engaged in Prevention of Mother-to-Child Transmission (PMTCT) Programs: A Commentary on the INSPIRE Studies in Malawi, Nigeria, and Zimbabwe. JAIDS Journal of Acquired Immune Deficiency Syndromes. 2017;75(2):S224–32. doi: 10.1097/qai.0000000000001375 28498193

[6] Phiri S, Tweya H, van Lettow M, Rosenberg NE, Trapence C, Kapito-Tembo A, et al. Impact of facility- and community-based peer support models on maternal uptake and retention in Malawi’s Option B HIV prevention of mother-to-child transmission program: A 3-arm cluster randomized controlled trial (PURE Malawi). J Acquir Immune Defic Syndr. 2017;75(Suppl 2):S140–8. 10.1097/QAI.0000000000001357 28498183

[7] Fayorsey RN, Wang C, Chege D, Reidy W, Syengo M, Owino SO, et al. Effectiveness of a Lay Counselor-Led Combination Intervention for Retention of Mothers and Infants in HIV Care: A Randomized Trial in Kenya. J Acquir Immune Defic Syndr. 2019;80(1):56–63. doi: 10.1097/QAI.0000000000001882 30399035 PMC6319592

[8] Abuogi LL, Onono M, Odeny TA, Owuor K, Helova A, Hampanda K, et al. Effects of behavioural interventions on postpartum retention and adherence among women with HIV on lifelong ART: the results of a cluster randomized trial in Kenya (the MOTIVATE trial). J Int AIDS Soc. 2022;25(1):e25852. doi: 10.1002/jia2.25852 35041776 PMC8765560

[9] Carlucci JG, Yu Z, González P, Bravo M, Amorim G, das Felicidades Cugara C, et al. The effect of a Mentor Mothers program on prevention of vertical transmission of HIV outcomes in Zambézia Province, Mozambique: a retrospective interrupted time series analysis. J Int AIDS Soc. 2022;25(6):e25952. doi: 10.1002/jia2.25952 35718940 PMC9207359

[10] National AIDS and STI Control Program (NASCOP). National Guidelines for PMTCT Peer Education and Psychosocial Support in Kenya: The Kenya Mentor Mother Program. Nairobi, Kenya: Ministry of Health, Kenya. 2012. http://guidelines.health.go.ke:8000/media/National_Guidelines__for__PMTCT_Peer_Education_and_Psychosocial_Support_in_Kenya_KMMP.pdf

[11] Onono M, Odwar T, Abuogi L, Owuor K, Helova A, Bukusi E, et al. Effects of Depression, Stigma and Intimate Partner Violence on Postpartum Women’s Adherence and Engagement in HIV Care in Kenya. AIDS Behav. 2020;24(6):1807–15. doi: 10.1007/s10461-019-02750-y 31813076 PMC7228848

[12] Humphrey J, Wanjama E, Carlucci JG, Naanyu V, Were E, Muli L, et al. Preferences of Pregnant and Postpartum Women for Differentiated Service Delivery in Kenya. J Acquir Immune Defic Syndr. 2023;94(5):429–36. doi: 10.1097/QAI.0000000000003303 37949446 PMC10642693

[13] Humphrey J, Wanjama E, Carlucci JG, Naanyu V, Muli L, Were E, et al. Understanding Women’s Preferences for Prevention of Mother-to-Child HIV Transmission Services in Kenya. Int J MCH AIDS. 2024;13:e009. doi: 10.25259/IJMA_6_2024 38840934 PMC11152576

[14] AMPATH Global. https://www.ampathglobal.org/

[15] AMPATH-Kenya: Where we work. [Available from: https://www.ampathkenya.org/where-we-work

[16] Goggin K, Hurley EA, Staggs VS, Wexler C, Nazir N, Gautney B, et al. Rates and Predictors of HIV-Exposed Infants Lost to Follow-Up During Early Infant Diagnosis Services in Kenya. AIDS Patient Care STDS. 2019;33(8):346–53. doi: 10.1089/apc.2019.0050 31369296 PMC6661912

[17] Braitstein P, Songok J, Vreeman RC, Wools-Kaloustian KK, Koskei P, Walusuna L, et al. “Wamepotea” (they have become lost): outcomes of HIV-positive and HIV-exposed children lost to follow-up from a large HIV treatment program in western Kenya. J Acquir Immune Defic Syndr. 2011;57(3):e40–6. doi: 10.1097/QAI.0b013e3182167f0d 21407085 PMC3145828

[18] Hodgson I, Plummer ML, Konopka SN, Colvin CJ, Jonas E, Albertini J, et al. A systematic review of individual and contextual factors affecting ART initiation, adherence, and retention for HIV-infected pregnant and postpartum women. PLoS One. 2014;9(11):e111421. doi: 10.1371/journal.pone.0111421 25372479 PMC4221025

[19] Phillips T, Thebus E, Bekker L-G, Mcintyre J, Abrams EJ, Myer L. Disengagement of HIV-positive pregnant and postpartum women from antiretroviral therapy services: a cohort study. J Int AIDS Soc. 2014;17(1):19242. doi: 10.7448/IAS.17.1.19242 25301494 PMC4192834

[20] Sam-Agudu NA, Ramadhani HO, Isah C, Erekaha S, Fan-Osuala C, Anaba U, et al. The Impact of Structured Mentor Mother Programs on Presentation for Early Infant Diagnosis Testing in Rural North-Central Nigeria: A Prospective Paired Cohort Study. J Acquir Immune Defic Syndr. 2017;75 Suppl 2:S182–9. doi: 10.1097/QAI.0000000000001345 28498188

[21] Feldstein AC, Glasgow RE. A practical, robust implementation and sustainability model (PRISM) for integrating research findings into practice. Jt Comm J Qual Patient Saf. 2008;34(4):228–43. doi: 10.1016/s1553-7250(08)34030-6 18468362

[22] McCreight MS, Rabin BA, Glasgow RE, Ayele RA, Leonard CA, Gilmartin HM, et al. Using the Practical, Robust Implementation and Sustainability Model (PRISM) to qualitatively assess multilevel contextual factors to help plan, implement, evaluate, and disseminate health services programs. Transl Behav Med. 2019;9(6):1002–11. doi: 10.1093/tbm/ibz085 31170296

[23] Grove RW. An analysis of the constant comparative method. International Journal of Qualitative Studies in Education. 1988;1(3):273–9. doi: 10.1080/0951839900030105a

[24] Tong A, Sainsbury P, Craig J. Consolidated criteria for reporting qualitative research (COREQ): a 32-item checklist for interviews and focus groups. Int J Qual Health Care. 2007;19(6):349–57. doi: 10.1093/intqhc/mzm042 17872937

[25] National AIDS and STI Control Programme (NASCOP) K. Improving the quality and efficiency of health services in Kenya: A practical handbook for HIV managers and service providers on differentiated care. Nairobi. 2016.

[26] National AIDS and STI Control Programme (NASCOP) Kenya. Guidelines on use of antiretroviral drugs for treating and preventing HIV infection in Kenya. Nairobi: National AIDS and STI Control Programme (NASCOP) Kenya. 2016.

[27] World Health Organization. HIV Treatment and Care: What’s New in Service Delivery. Geneva; 2015.

[28] World Health Organization. Key considerations for differentiated antiretroviral therapy delivery for specific populations: children, adolescents, pregnant and breastfeeding women and key populations. Geneva: World Health Organization. 2017.

[29] National AIDS & STI Control Program Ministry of Health Kenya. Kenya HIV Prevention and Treatment Guidelines. 2022.

[30] Hagey JM, Li X, Barr-Walker J, Penner J, Kadima J, Oyaro P, et al. Differentiated HIV care in sub-Saharan Africa: a scoping review to inform antiretroviral therapy provision for stable HIV-infected individuals in Kenya. AIDS Care. 2018;30(12):1477–87. doi: 10.1080/09540121.2018.1500995 30037312

[31] Humphrey J, Kipchumba B, Carlucci JG, Midiwo R, Were E, McGuire A, et al. Human-centered design of an adapted differentiated service delivery model for pregnant and postpartum women living with HIV in Kenya. AIDS Care. 2025;37(9):1434–49. doi: 10.1080/09540121.2025.2485397 40168585 PMC12353083

[32] Humphrey J, Carlucci JG, Wanjama EK, Naanyu V, Muli L, Alera JM, et al. Implementing WHO Differentiated Service Delivery Model for Pregnant and Breastfeeding Women and Infants Living with HIV: Insights from Kenyan Healthcare Providers. Int J MCH AIDS. 2025;14:e004. doi: 10.25259/IJMA_43_2024 40046033 PMC11878722

[33] Humphrey JBG, Kipchumba B, Musick B, Sang E, Carlucci JG, Were E, et al. Improved retention after implementing differentiated services for postpartum women with HIV in Kenya. In: Conference on Retroviruses and Opportunistic Infections; February 22-25; Denver, Colorado, 2026.

[34] Adeniyi A, Ikemeri JE, Chelagat S, Kasaya GA, Jumah A, Maldonado LY, et al. Women’s economic empowerment through chamas: Evidence from a maternal health program in Kenya. Afr J Reprod Health. 2026;30(3):13–23. doi: 10.29063/ajrh2026/v30i3.2 41697064

[35] Genberg BL, Wilson-Barthes MG, Omodi V, Hogan JW, Steingrimsson J, Wachira J, et al. Microfinance, retention in care, and mortality among patients enrolled in HIV care in East Africa. AIDS. 2021;35(12):1997–2005. doi: 10.1097/QAD.0000000000002987 34115646 PMC8963387

[36] Maldonado LY, Bone J, Scanlon ML, Anusu G, Chelagat S, Jumah A, et al. Improving maternal, newborn and child health outcomes through a community-based women’s health education program: a cluster randomised controlled trial in western Kenya. BMJ Glob Health. 2020;5(12):e003370. doi: 10.1136/bmjgh-2020-003370 33293295 PMC7725102

[37] Maldonado LY, Songok JJ, Snelgrove JW, Ochieng CB, Chelagat S, Ikemeri JE, et al. Promoting positive maternal, newborn, and child health behaviors through a group-based health education and microfinance program: a prospective matched cohort study in western Kenya. BMC Pregnancy Childbirth. 2020;20(1):288. doi: 10.1186/s12884-020-02978-w 32398156 PMC7216653

[38] Doukani A, van Dalen R, Valev H, Njenga A, Sera F, Chibanda D. A community health volunteer delivered problem-solving therapy mobile application based on the Friendship Bench “Inuka Coaching” in Kenya: A pilot cohort study. Glob Ment Health (Camb). 2021;8:e9. doi: 10.1017/gmh.2021.3 34026239 PMC8127638

[39] Brooks MJ, MacLean SA, Eschliman EL, Poku OB, Molebatsi K, Ahmed C. Piloting a Near-Peer Lay Counselor Based Problem Solving Therapy Intervention for Youth With and Without HIV in Botswana: An Adaptation of the Friendship Bench. J Adolesc Health. 2026. 10.1016/j.jadohealth.2025.12.260 PMC13175587 41653177

[40] Simms V, Abas MA, Müller M, Munetsi E, Dzapasi L, Weiss HA, et al. Effect of a brief psychological intervention for common mental disorders on HIV viral suppression: A non-randomised controlled study of the Friendship Bench in Zimbabwe. PLOS Glob Public Health. 2024;4(1):e0001492. doi: 10.1371/journal.pgph.0001492 38236786 PMC10796049

[41] Bengtson AM, Filipowicz TR, Mphonda S, Udedi M, Kulisewa K, Meltzer-Brody S, et al. An Intervention to Improve Mental Health and HIV Care Engagement Among Perinatal Women in Malawi: A Pilot Randomized Controlled Trial. AIDS Behav. 2023;27(11):3559–70. doi: 10.1007/s10461-023-04070-8 37084104 PMC10119837

[42] Wogrin C, Willis N, Mutsinze A, Chinoda S, Verhey R, Chibanda D, et al. It helps to talk: A guiding framework (TRUST) for peer support in delivering mental health care for adolescents living with HIV. PLoS One. 2021;16(3):e0248018. doi: 10.1371/journal.pone.0248018 33657185 PMC7928463

[43] Barnett ML, Puffer ES, Ng LC, Jaguga F. Effective training practices for non-specialist providers to promote high-quality mental health intervention delivery: A narrative review with four case studies from Kenya, Ethiopia, and the United States. Glob Ment Health (Camb). 2023;10:e26. doi: 10.1017/gmh.2023.19 37854408 PMC10579690

[44] Giusto A, Vander Missen MR, Kosgei G, Njiriri F, Puffer E, Kamaru Kwobah E, et al. Peer-delivered Problem-solving Therapy for Adolescent Mental Health in Kenya: Adaptation for Context and Training of Peer-counselors. Res Child Adolesc Psychopathol. 2023;51(9):1243–56. doi: 10.1007/s10802-023-01075-8 37219680 PMC10203666

[45] Jaguga F, Kiburi SK, Temet E, Aalsma MC, Ott MA, Maina RW, et al. A scoping review of substance use brief interventions in Africa. PLOS Glob Public Health. 2024;4(10):e0003340. doi: 10.1371/journal.pgph.0003340 39446874 PMC11501030

