# Supplementary material for: Perspectives on enhancing peer support services for mothers living with HIV in Kenya: A qualitative assessment of Mentor Mothers and the mothers they serve
Source: PLOS Glob Public Health. 2026 Sep 18;6(9):e0007277. doi: 10.1371/journal.pgph.0007277 (PMC13588416; doi:10.1371/journal.pgph.0007277)
Supplement: Supplementary file 1 [file pgph.0007277.s001.docx]

**In-Depth Interview Guide**

**Introduction:**

Thank you for agreeing to take part in this research study. My name is [*name*], and I am part of the research team. We are interested in better understanding your perceptions of Mentor Mother peer support services for pregnant and postpartum women living with HIV. We also want to hear your opinions about how Mentor Mother services might be modified to promote better outcomes for women and their babies.

There are no right or wrong answers to the questions. Your responses will be audio recorded, and the recordings will be analyzed to better understand your responses. We will not write your name on the paper with your answers on it or use it in the audio recording. This way only the study team will know your answers and their link to your identifying information. You do not have to answer questions you do not want to answer. If you feel uncomfortable at any point, please let us know; we can either move on to the next question or stop the interview altogether.

Once again, we want to thank you for agreeing to be part of this study and for your willingness to share your experiences and perspectives with us.

**Brief Questionnaire:**

To start, we would like to gather some background information. Again, you can choose to skip any questions that you prefer not to answer.

| **For women living with HIV (WLHIV)** [*for Mentor Mothers, skip to Q13.*] | |
| --- | --- |
| Q1. What is your age? | 🞎 ____ years  🞎 Prefer not to answer |
| Q2. How many children do you have? | 🞎 ____  🞎 Prefer not to answer |
| Q3. How old is your youngest child? | 🞎 ____ years, ____months  🞎 Prefer not to answer |
| Q4. How old is your oldest child? | 🞎 ____ years, ____months  🞎 Not applicable [*only 1 child; same as Q3., above*]  🞎 Prefer not to answer |
| Q5. When were you diagnosed with HIV? | 🞎 During my most recent pregnancy  🞎 Prior to my most recent pregnancy, but within the past 2 years  🞎 More than 2 years ago  🞎 Not sure  🞎 Prefer not to answer |
| Q6. Where do you live most of the time? [ *only record primary place of residence* ] | 🞎 In the city [*i.e., urban*]  🞎 On the outskirts of the city [*i.e., peri-urban*]  🞎 In a rural area  🞎 Not sure  🞎 Prefer not to answer |
| Q7. Including you, your children, and other adults, how many people live in your home? | 🞎 ____  🞎 Prefer not to answer |
| Q8. Do the other adults in your home know your HIV status? | 🞎 Yes, all adults know  🞎 Yes, some adults know  🞎 No  🞎 Not sure  🞎 Not applicable [*no other adults*]  🞎 Prefer not to answer |
| Q9. Do you have a male partner (e.g., husband, fiancé, boyfriend)? | 🞎 Yes  🞎 No  🞎 Prefer not to answer |
| Q10. Does your male partner know your HIV status? | 🞎 Yes  🞎 No  🞎 Not sure  🞎 Not applicable [no male partner; *“No” to Q9., above*]  🞎 Prefer not to answer |
| Q11. What is your highest level of education? | 🞎 Professional  🞎 University / post-secondary  🞎 Secondary  🞎 Primary  🞎 None  🞎 Prefer not to answer |
| Q12. Do you have a phone? | 🞎 Yes, my own  🞎 Yes, shared with another person in my home/community  🞎 No  🞎 Prefer not to answer |
| **For Mentor Mothers** [*for WLHIV, skip to Q18.*] | |
| Q13. What is your age? | 🞎 ____ years  🞎 Prefer not to answer |
| Q14. How long have you been a Mentor Mother? | 🞎 ____ years, ____months  🞎 Prefer not to answer |
| Q15. Where do you provide Mentor Mother services? [*check all that apply*] | 🞎 Antenatal clinic  🞎 Postnatal clinic  🞎 Maternity ward  🞎 In the community [*i.e., home visits*]  🞎 Other: _____­­­­____________  ­­­­_______________________  ­­­_______________________  🞎 Prefer not to answer |
| Q16. Before becoming a Mentor Mother, did you have any experience working in a healthcare setting? | 🞎 No  🞎 Yes, explain: ­­­­____________  ­­­­_______________________  ­­­_______________________  🞎 Prefer not to answer |
| Q17. What is your highest level of education? | 🞎 Professional  🞎 University / post-secondary  🞎 Secondary  🞎 Primary  🞎 None  🞎 Prefer not to answer |

**Current Services:**

Q18. (Insert here a succinct description of the mentor mothers program)

Can you tell me about your experiences with the Mentor Mother program?

***Probes for WLHIV:***

a. This might include experiences of being a patient/client.
b. What has your experience with the Mentor Mothers program been like?
c. What is your relationship with the Mentor Mothers like? Do the Mentor Mothers know you and your baby well? Is this relationship important to you?
d. Has your perception of the Mentor Mother program changed over time?

***Probes for Mentor Mothers:***

a. Describe to me your work as a Mentor Mother. What is a typical day like?
b. This might include experiences working with clients, your experiences working at the health clinics,
c. What has your experience working as a Mentor Mother been like?
d. What is your relationship with your clients like? How well do you get to know your clients and their babies? Is this relationship important to you?
e. How is your relationship with other staff at the clinic? Do they value the services you provide as a Mentor Mother? Do you work well together?
f. Do you feel supported in your work as Mentor Mother? Supported by other Mentor Mothers? Supported by other staff at the clinic?
g. Is the Mentor Mothers program structured in a way that makes it easy to do your job? If so, how? If not, what are sort of things make it difficult to do your job?
h. Has your perception of the Mentor Mother program changed over time?

Q19. From your perspective, can you tell me how the Mentor Mother program currently works?

***Probes for WLHIV:***

a. How often do you meet with a Mentor Mother? Are there circumstances where you would meet with a Mentor Mother more or less frequently? Antepartum? Postpartum?
b. Do you always meet with the same Mentor Mother, or do you meet with various Mentor Mothers depending on the day?
c. Where do you meet with the Mentor Mother? Are these interactions one-on-one? Are you able to speak freely?
d. What kinds of things do you discuss with the Mentor Mother? Are there set things you talk about at every visit? Are the things you talk about always relevant to your and your baby’s situation? Are you able to bring up and discuss specific questions or needs that you might have?
e. What are Mentor Mothers able to do for you and other clients? Have you received any of these services from Mentor Mothers? If so, please tell me about one of those experiences, and whether you were satisfied with the service provided.
f. Has a Mentor Mother ever called you on your phone or sent you a text/SMS message? If so, why? If not, would you be open to receiving calls/SMS from a Mentor Mother? Why or why not?
g. Has a Mentor Mother ever visited you at your home or somewhere else in the community? If so, why? If not, would be open to meeting a Mentor Mother at your home or elsewhere in the community? Why or why not?
h. From your perspective, do Mentor Mothers work with other clinic staff to identify and address the needs of clients? If so, can you give an example. If not, how might this be improved?

***Probes for Mentor Mothers:***

a. How often do you meet with clients? Are there circumstances where you would meet with a client more or less frequently? Antepartum? Postpartum?
b. Where do you meet with clients? Are these interactions one-on-one? Are you and clients able to speak freely?
c. What kinds of things do you discuss with clients? Are there topics you are supposed to discuss at every visit? Are there topics that you discuss only at certain visits?
d. Are the things you talk about always relevant to your client’s or their baby’s specific situation? Are you able identify and discuss specific issues or needs that your client or their baby might have? If so, please tell me about one of those experiences, and whether you were satisfied with your ability to address their issue.
e. Have you ever called a client on their phone or sent them a text/SMS message? If so, why? If not, would you be open to calling or texting your clients? Why or why not?
f. Have you ever visited a client in their home or somewhere else in the community? If so, why? If not, would you be open meeting a client in their home or elsewhere in the community? Why or why not?
g. From your perspective, do Mentor Mothers work with other clinic staff to identify and address the needs of clients? If so, can you give an example? If not, how might this be improved?

Q20. Are there things you like about the Mentor Mothers program?

***Probes for WLHIV:***

a. Do you perceive any benefits to participating in the Mentor Mothers program?
b. Is it convenient and helpful for you to meet with a Mentor Mother?
c. If the Mentor Mothers program went away, what would you miss the most?
d. Are there things that Mentor Mothers currently do for you or other clients, that you wish they would do more?
e. Has support from a Mentor Mother been helpful for you and/or your baby? If so, can you tell me about a time when the Mentor Mother program was helpful for you and/or your baby?

***Probes for Mentor Mothers:***

a. Do you think clients benefit from participating in the Mentor Mothers programs?
b. Is it convenient and helpful for clients to meet with a Mentor Mother?
c. If the Mentor Mothers program went away, what would clients be missing out on?
d. Are there services that you currently provide for clients that you wish you could do more?
e. Can you think of a time when you were able to support a client and/or her baby? If so, can you tell me about how the Mentor Mother program was helpful in that situation?

Q21. Are there things you dislike about the Mentor Mothers program?

***Probes for WLHIV:***

a. Do you perceive any downsides to participating in the Mentor Mothers program? If so, please elaborate.
b. Is it ever inconvenient or burdensome for you to meet with a Mentor Mother? If so, please elaborate.
c. Are there services that Mentor Mothers currently provide that you feel are unnecessary?
d. Can you tell me about a time when you were unhappy with some aspect of the Mentor Mothers program?
e. Have you ever had a negative interaction with a Mentor Mother? If so, can you please share some details about that experience?
f. Has the Mentor Mothers program ever failed to meet your expectations? If so, can you share an example of when the program was unhelpful to you and/or your baby?
g. If you were to change one thing about the Mentor Mothers program, what would it be?

***Probes for Mentor Mothers:***

a. Do you perceive any downsides to clients participating in the Mentor Mothers program? Do clients ever seem inconvenienced or unhappy to meet with a Mentor Mother? Can you tell me about a time when a client was unhappy with some aspect of the Mentor Mothers program?
b. Have you ever had a negative interaction with a client? If so, can you please share some details about that experience?
c. Are there current Mentor Mother services that you feel are unwanted or unnecessary and could be done away with?
d. Are the Mentor Mother services you wish you could provide but that you are currently unable to provide?
e. If you were to change one thing about the Mentor Mothers program, what would it be?

**Desires for Additional Services / Proposed Modifications to the Current Strategy:**

Q22. If you could make changes to the Mentor Mother program, what would you recommend?

***Probes for WLHIV:***

a. Are there additional services that you like to receive from Mentor Mothers? During antennal care? During postnatal care?
b. Are there services that Mentor Mothers currently provide that you would prefer not to receive or that you would somehow change? During antennal care? During postnatal care?
c. Are there Mentor Mother services that could be provided more efficiently? For example, providing certain services only to those who need them, but not providing those services to others who might not need them.
d. Are there particular topics that you would like more education or counselling about from a Mentor Mother?
e. Would you change the location where Mentor Mother services are provided? If yes, where should they be located?
f. Would you change the frequency of meetings with a Mentor Mother? Which meetings should be less frequent? More frequent? During antennal care? During postnatal care?

***Probes for Mentor Mothers:***

a. Are there additional services that you like to provide to your clients? During antennal care? During postnatal care?
b. Are there services that you currently provide to clients that you think are unnecessary or that you would somehow change? During antennal care? During postnatal care?
c. Are there Mentor Mother services that could be provided more efficiently? For example, providing certain services only to those who need them, but not providing those services to others who might not need them.
d. Are there particular topics that you think would be of benefit to clients? For example, coping with a new HIV diagnosis, disclosure of HIV status to family members, strategies for improving adherence to medical advice, managing postpartum depression, etc. Would you need additional education, training, or resources/materials to provide this sort of education or counselling to your clients?
e. Would you change the location where Mentor Mother services are provided? Elsewhere in the clinic? Outside the clinic? In your home?
f. Would you change the frequency of meetings with a Mentor Mother? Less frequent? More frequent? During antennal care? During postnatal care?
g. If you proposed any changes/recommendations (as above), would these be feasible in the context of current resource constraints, or would additional resources be needed? Probe for each proposed change separately

Q23. What do you think about the idea of modifying the Mentor Mother program such that some services are allocated based on a client’s specific needs and risk factors?

***Probes for WLHIV:***

a. For example, women with a recent HIV diagnosis might receive additional counselling from a Mentor Mother about the importance and process of disclosing their HIV status to their intimate partner, while those who have a longstanding HIV diagnosis and who have already disclosed to their HIV status to the their partner would not receive this additional counselling.
b. Another example might be providing more frequent follow up visits or phone calls for women struggling to keep their viral load suppressed, while those with a long track record of viral suppression might have less frequent encounters with a Mentor Mother.
c. Would you be interested in a more tailored approach like this? Why or why not?
d. Other than the examples provided (above), are there other Mentor Mother services that you think could be provided to some clients based on their needs/risk factors?
e. Likewise, are there Mentor Mother services that you think could be omitted for some clients based on their specific situation/lack of risk factors?
f. Would you be comfortable with Mentor Mothers or other clinic staff asking you additional questions, some of which might be personal, in order to determine which services you should or should not receive?

***Probes for Mentor Mothers:***

a. For example, women with a recent HIV diagnosis might receive additional counselling from a Mentor Mother about the importance and process of disclosing their HIV status to their intimate partner, while those who have a longstanding HIV diagnosis and who have already disclosed to their HIV status to the their partner would not receive this additional counselling.
b. Another example might be providing more frequent follow up visits or phone calls for women struggling to keep their viral load suppressed, while those with a long track record of viral suppression might have less frequent encounters with a Mentor Mother.
c. Would you be interested in a more tailored approach like this? Why or why not?
d. Other than the examples provided (above), are there other Mentor Mother services that you think could be provided to some clients based on their needs/risk factors?
e. Likewise, are there Mentor Mother services that you think could be omitted for some clients based on their specific situation/lack of risk factors?
f. In your opinion, what would be the best approach to identify client needs/risk factors (or lack thereof)? As a Mentor Mother, would you be comfortable asking additional questions to identify patient risk factors?

As a Mentor Mother, would you be comfortable accessing medical records (e.g., looking at viral load results in POC)?

g. Or would this task be better suited for a clinician or other clinic staff?
h. Would a “decision support” tool embedded in the workflow be helpful? If so, what might this look like and how might it be implemented?

**Barriers and Facilitators:**

Q24. If the Mentor Mother program was modified such that some services are allocated based on a client’s specific needs/risk factors, can you think of any barriers to being able to identify or address those needs?

***Probes for WLHIV:***

a. Would you or other clients be willing to share personal information with the Mentor Mothers or other clinic staff? Would you be willing to share some types of information, but not others? What sort of information would be hesitant or unwilling to share?
b. Do you feel like the Mentor Mothers are respectful of your privacy and that they strive to protect your confidentiality? Have you ever withheld private information from the Mentor Mother? If so, why? Would you be more willing to share private information with a clinician or other clinic staff?
c. Would engaging your male partner help or hinder you from receiving Mentor Mother support? For some issues but not others? With regards to phone calls, text messages/SMS, and/or home visits?
d. Would engaging a close family member (e.g., your mother or mother-in-law), or some other treatment supporter help or hinder you from receiving Mentor Mother support? For some issues but not others? With regards to phone calls, text messages/SMS, and/or home visits?
f. Are there some issues/needs/risk factors that might interfere with a client’s ability to successfully navigate HIV care and treatment for themselves and their baby, but that are beyond the scope of what a Mentor Mother could address? For example, work obligations, seasonal travel, financial problems, other health problems, etc.
g. Which clients might find it easier to engage in problem-focused Mentor Mother services? For example, higher levels or education/literacy, supportive family and friends, living close to the clinic, etc.

***Probes for Mentor Mothers:***

a. Do you think clients be willing to share personal information with Mentor Mothers or other clinic staff? Might they share some types of information, but not others? What sort of information might they be hesitant or unwilling to share?
b. Would engaging male partners, close family members (e.g., mother or mother-in-law), or some other treatment supporter potentially help or hinder clients from receiving Mentor Mother support? For some issues but not others? With regards to phone calls, text messages/SMS, and/or home visits?
c. Are there some issues/needs/risk factors that might interfere with a client’s ability to successfully navigate HIV care and treatment for themselves and their baby, but that are beyond the scope of what a Mentor Mother could address? For example, work obligations, seasonal travel, financial problems, other health problems, etc.
d. Are there some client characteristics that might make it easier for clients to engage in problem-focused Mentor Mother services? For example, higher levels or education/literacy, supportive family and friends, living close to the clinic, etc.
e. Are there any issues with how patients “flow” through the clinic, that might make it challenging to identify or address client needs?
f. If an issue requiring referral to another clinic (e.g., severe postpartum depression) was identified, do you envision any barriers to linking clients to that additional care? E.g. Fees? Transport?
g. What other resources can be leveraged help address these barriers?

Q25. If changes to the current Mentor Mother strategy were proposed (e.g., if the Mentor Mother program was modified such that some services are allocated based on a client’s specific needs/risk factors), what additional resources (if any) would be needed? [*for Mentor Mothers only; skip for WLHIV*]

***Probes for Mentor Mothers:***

a. [*Obviously, responses to these questions will depend on the type of changes proposed, as well as respondents’ responses to previous questions. That said, please solicit both general opinions and ideas that specifically relate to prior responses.*]
b. Would additional staff be needed? Please elaborate, in general and based on your responses to previous questions.
c. Would additional training(s) be needed for MMs? If so, what sort of trainings would be most useful, and how frequently would they need to be repeated? Do you already have some training or experience that you might be able to leverage in your work as a Mentor Mother? Would you be willing or able to train others?
d. Are there already other clinic staff or clinic processes that could be utilized to avoid burdening Mentor Mothers with additional tasks? In other words, are there ways to identify and address client needs that would complement a modified Mentor Mother strategy, rather than require a lot of extra effort from Mentor Mothers?
e. Are there other clinic-based or community resources that could be leveraged to enhance the Mentor Mother program and/or to address identifiable client needs? If so, what are the quality and availability of these other resources? What things might make it easier or harder to integrate these resources with a modified Mentor Mother strategy?
f. Would additional pay, or other incentives, make it easier or more acceptable for Mentor Mothers to implement changes to the current strategy?
g. What else would be needed to ensure high-quality and sustainable implementation of a modified Mentor Mother strategy?

Q26. If changes to the current Mentor Mother strategy were proposed (e.g., if the Mentor Mother program was modified such that some services are allocated based on a client’s specific needs/risk factors), would key staff be ready and willing to implement those changes? [*for Mentor Mothers only; skip for WLHIV*]

***Probes for Mentor Mothers:***

a. Would Mentor Mothers be supportive of and ready for change? Why or why not?
b. Would clinic staff be supportive of and ready for change? Why or why not?
c. Would PMTCT program leaders be supportive of and ready for change? Why or why not?
d. If you or other staff are hesitant or unwilling to make changes to the current Mentor Mother strategy, do you have ideas about how to compromise or overcome these concerns?
e. Are there other stakeholders whose perspectives should be considered?

This is the end of the interview. Are there any final thoughts or comments you would like to share? Thank you so much for sharing your perspectives and your time with us.

**FIELD NOTES**

**Interviewer name:**

**Venue:**

**Date:**

**IDI #:**

**Interviewer Notes:**

a. Descriptive notes

____________________________________________________________________

____________________________________________________________________

____________________________________________________________________

____________________________________________________________________

____________________________________________________________________

____________________________________________________________________

____________________________________________________________________

____________________________________________________________________

____________________________________________________________________

____________________________________________________________________

____________________________________________________________________

____________________________________________________________________

____________________________________________________________________

____________________________________________________________________

____________________________________________________________________

____________________________________________________________________

____________________________________________________________________

b. Reflective notes

____________________________________________________________________

____________________________________________________________________

____________________________________________________________________

____________________________________________________________________

____________________________________________________________________

____________________________________________________________________

____________________________________________________________________

____________________________________________________________________

____________________________________________________________________

____________________________________________________________________

____________________________________________________________________

____________________________________________________________________

____________________________________________________________________

____________________________________________________________________

___________________________________________________________________

____________________________________________________________________

____________________________________________________________________
