# Supplementary material for: Perspectives on enhancing peer support services for mothers living with HIV in Kenya: A qualitative assessment of Mentor Mothers and the mothers they serve
Source: PLOS Glob Public Health. 2026 Sep 18;6(9):e0007277. doi: 10.1371/journal.pgph.0007277 (PMC13588416; doi:10.1371/journal.pgph.0007277)
Supplement: Supplementary file 2 [file pgph.0007277.s002.docx]

Inclusivity in global research

PLOS’ policy on inclusivity in global research aims to improve transparency in the reporting of research performed outside of researchers’ own country or community and ensures that PLOS publications reporting global research adhere to high standards for research ethics and authorship. Authors of relevant research articles may be asked to complete the questionnaire below, which outlines ethical, cultural, and scientific considerations specific to inclusivity in global research. This questionnaire may be requested when researchers have travelled to a different country to conduct research, if research uses samples collected in another country, research with Indigenous populations or their lands, or if research is on cultural artefacts. Researchers travelling to another country solely to use laboratory equipment will not normally be required to complete the questionnaire. However, the questionnaire can be requested at the journal’s discretion for any submission – if you have been requested to complete this questionnaire by the PLOS journal you submitted to, please do so.

Please complete the questionnaire below and include this as a Supporting Information file with your manuscript. Note that if your paper is accepted for publication, this checklist will be published with your article in the supporting information files. Please ensure that you reference the checklist in the main body of your manuscript. We suggest adding a subsection ‘Inclusivity in global research’ to your Methods section and adding the following sentence: “Additional information regarding the ethical, cultural, and scientific considerations specific to inclusivity in global research is included in the Supporting Information (SX Checklist)”

The questions have been designed to be applicable to a wide range of study types, and there are subsections for both human subjects research and non-human subjects research. If any of the questions are not relevant to your research please mark them as “N/A” as appropriate.

**Ethical considerations, permits and authorship**

*This section is applicable to all research types.*

Provide details as to who granted permissions and/or consent for the study to take place in the Methods section of your manuscript. This should include the names of **all** ethics boards, governmental organizations, community leaders or other bodies that provided approval for the study. If individuals provided approval refer to these people by their role or title but do not list their name(s).

This information can be found in the Ethics Approvals section on page 8.

If there were any deviations from the study protocol after approval was obtained please provide details of these changes in the Methods section of your manuscript.
Did this study involve local collaborators that are residents of the country where the research was conducted or members of the community studied? If you do not have any authors from said communities, please provide an explanation for this below.

N/A

The following co-authors are residents of Kenya, where the research was conducted: Rosa Chemwey Ndiema, Violet Naanyu, Marylydia Atieno Kiano, and Edwin Were. Additionally, Eunice Mwangi and Lindah Muli, Kenyan Research Assistants not meeting authorship requirements, are cited in the Acknowldegemetns section for their assistance with study logistics and participant recruitment and engagement.

Everyone listed as an author should meet PLOS’ criteria for authorship and all individuals who meet these criteria should be included in the author byline, rather than the acknowledgements. For further information please see the journal’s Authorship Policy.

**Human subjects research (e.g. health research, medical research, cross-cultural psychology)**

Did you obtain written informed consent from a representative of the local community or region before the research took place? How did you establish who speaks for the community? Details of written informed consent obtained from study participants should be reported separately in the Methods section of your manuscript.

Details of written informed consent obtained from study participants is reported in the Methods section of the manuscript on pages 5 and 7. No additional written informed consent from a representative of the local community or region was obtained before the research took place; however, county- and clinic-level representatives from the Kenya Ministry of Health granted us permission to carry out this research with participants from each of the three health facilities included in this study, as is customary for the well-established research program embedded within AMPATH (Academic Model Supporting Access to Healthcare)-supported public health facilities in this region. And, to be clear, we did not ask participants to “speak for the community”; rather, participants were asked to provide their unique individual perspectives, and data saturation was achieved through this approach.

How did members of the local community provide input on the aims of the research investigation, its methodology, and its anticipated outcome(s)?

From inception through all stages of this research, input from the local community has been actively sought and integrated to inform the study aims, methodology, and anticipated outcomes. The research itself was motivated by sustained engagement with mothers living with HIV and the clinical teams who serve them. Community input has been incorporated through multiple channels, including individual interactions during clinical care, structured workshops and listening sessions with Mentor Mothers and clinicians providing prevention of mother-to-child transmission (PMTCT) services, and ongoing collaboration with Kenyan partners whose perspectives and priorities are central to the research. These engagements have directly shaped the research questions, design, and intended impact.

When engaging with the local community, how did you ensure that the informed consent documents and other materials could be understood by local stakeholders?

Informed consent documents and qualitative interview guides were developed in English and translated into Kiswahili. Both language versions were pilot tested with local stakeholders to ensure clarity, cultural appropriateness, and comprehension prior to finalization. Feedback from these pilot activities informed revisions to simplify language and improve understanding. In addition, both the Moi University/Moi Teaching and Referral Hospital Institutional Research and Ethics Committee in Kenya and the Indiana University Institutional Review Board in the United States require that participant-facing materials be accessible to individuals with low levels of literacy. Their review and approval provided an additional layer of assurance that materials were clear, appropriate, and understandable.

Will the findings of the research be made available in an understandable format to stakeholders in the community where the study was conducted (e.g. via a presentation, summary report, copies of publications, etc.)? Please provide details of how this will be achieved.

Findings from this research have already been shared with clinical teams at the three participating sites through on-site presentations and workshops. Copies of this and related manuscripts will also be provided to these stakeholders once published. In addition, we will disseminate findings to the communities from which participants were recruited using accessible, community-appropriate formats. Community leaders will be engaged to guide dissemination strategies, ensure messaging is culturally appropriate, and facilitate access. These activities will not only support understanding of study findings but also provide an opportunity to solicit community priorities for future research and foster ongoing stakeholder engagement.

**Non-human subjects research using specimens/ animals collected as part of the study, or those housed in archival collections. Examples include archaeology, paleontology, botany and zoology.**

Did the permission you obtained from a local authority to perform the study include an agreement on access to outputs and benefit sharing? This may include procedures to enable fair distribution of the benefits and resources arising from the research performed. Please include any details of Prior Informed Consent and Benefit Sharing Agreements obtained. These may be required by field-specific regulations, for example the Convention on Biological Diversity (CBD) and the associated Nagoya Protocol.

N/A

If the material used in your study was imported, please A) provide the year it was imported and B) indicate whether permits were obtained to import/export the materials used, C) provide details of any permits obtained. If this information is not available, please indicate this.

N/A

If you used archival specimens, please state how the material used in your study was acquired by the institute it is held in and provide details of any permits obtained for the original excavations/ sample collection. If this information is not available, please indicate this.

N/A

How was the potential cultural significance of the materials collected in your study to local communities considered in your research design? Were Indigenous peoples and/or local researchers and institutions involved with archaeological excavations / collection of specimens? If so, please provide a description of their involvement.

N/A

If your manuscript includes photographs of human remains please indicate whether authors obtained permission from descendants or affiliated cultural communities to do so.

N/A
